# Mother-to-Child HIV-1 Transmission Events Are Differentially Impacted by Breast Milk and Its Components from HIV-1-Infected Women

**DOI:** 10.1371/journal.pone.0145150

**Published:** 2015-12-17

**Authors:** Ruizhong Shen, Jenna Achenbach, Yue Shen, Jana Palaia, Jeremy T. Rahkola, Heidi J. Nick, Lesley E. Smythies, Michelle McConnell, Mary G. Fowler, Phillip D. Smith, Edward N. Janoff

**Affiliations:** 1 Department of Medicine (Division of Gastroenterology), University of Alabama at Birmingham, Birmingham, Alabama, United States of America; 2 Mucosal and Vaccine Research Program Colorado (MAVRC), University of Colorado Denver, Aurora, Colorado, United States of America; 3 Department of Biological Sciences, Auburn University, Auburn, Alabama United States of America; 4 Denver Veterans Affairs Medical Center, Denver, Colorado, United States of America; 5 Centers for Disease Control and Prevention, Atlanta, Georgia, United States of America; 6 The Makerere University-Johns Hopkins University Research Collaboration, Kampala, Uganda; 7 Veterans Affairs Medical Center, Birmingham, Alabama, United States of America; Vita-Salute San Raffaele University School of Medicine, ITALY

## Abstract

Breast milk is a vehicle of infection and source of protection in post-natal mother-to-child HIV-1 transmission (MTCT). Understanding the mechanism by which breast milk limits vertical transmission will provide critical insight into the design of preventive and therapeutic approaches to interrupt HIV-1 mucosal transmission. However, characterization of the inhibitory activity of breast milk in human intestinal mucosa, the portal of entry in postnatal MTCT, has been constrained by the limited availability of primary mucosal target cells and tissues to recapitulate mucosal transmission *ex vivo*. Here, we characterized the impact of skimmed breast milk, breast milk antibodies (Igs) and non-Ig components from HIV-1-infected Ugandan women on the major events of HIV-1 mucosal transmission using primary human intestinal cells and tissues. HIV-1-specific IgG antibodies and non-Ig components in breast milk inhibited the uptake of Ugandan HIV-1 isolates by primary human intestinal epithelial cells, viral replication in and transport of HIV-1- bearing dendritic cells through the human intestinal mucosa. Breast milk HIV-1-specific IgG and IgA, as well as innate factors, blocked the uptake and transport of HIV-1 through intestinal mucosa. Thus, breast milk components have distinct and complementary effects in reducing HIV-1 uptake, transport through and replication in the intestinal mucosa and, therefore, likely contribute to preventing postnatal HIV-1 transmission. Our data suggests that a successful preventive or therapeutic approach would require multiple immune factors acting at multiple steps in the HIV-1 mucosal transmission process.

## Introduction

Mother-to-child transmission (MTCT) of HIV-1 is a major public health problem in resource-limited countries, particularly those in Sub-Saharan Africa. Among the 350–500,000 annual cases of vertical transmissions, 25–44% are acquired after birth through HIV-1-infected breast milk ingested by nursing infants, who likely become infected when virus enters the mucosa of the small intestine [1, 2]. However, the majority of HIV-1-exposed breastfed infants (85–90%) do not become infected [2–6], despite daily exposure to the virus, suggesting that breast milk has the dual capability of mediating and blocking virus transmission, depending on the levels of innate factors, acquired immune factors, viral load, and other factors in breast milk [7–20]. Indeed, innate factors and HIV-1-specific antibodies in breast milk have been associated with reduced transmission of HIV-1 through breast milk [13–20]. Breast milk HIV-1-specific IgA response has been shown to correlate with reduced risk of postnatal MTCT of HIV-1 [20]. Breast milk, particularly the IgG component, can neutralize HIV-1 [21, 22]. In the non-antibody fraction of milk, innate factors such as defensins, mucins, and interleukin 15 have been associated with reduced breast milk transmission of HIV-1 and/or display HIV-1 inhibitory activity *in vitro* [13–18]. Recently, human breast milk from infected, as well as uninfected, women was shown to reduce HIV-1 transmission in humanized bone marrow/liver/thymus (BLT) mice [23, 24]. However, the effect of breast milk and its components on HIV-1 entry and infection in human intestinal mucosa, the portal of entry in postnatal MTCT [1, 2, 6], has not been defined. Moreover, most studies of the function of antibodies in breast milk in MTCT have focused on transcytosis, neutralization or antibody-dependent cell cytotoxicity using cell lines and PBMCs.

The upper gastrointestinal tract is the portal through which HIV-1 enters the host in the majority of MTCTs [1, 2, 6]. HIV-1 mucosal transmission involves three major events: (a) entry through or across the mucosal epithelium; (b) infection and subsequent replication in sub-epithelial mononuclear target cells; and (c) local dissemination and delivery of virus to draining lymph nodes to initiate systemic infection [25–27]. In the small intestine, transcytosis and translocation of HIV-1 by epithelial cells or surface-penetrating dendritic cells (DCs) are the likely cellular routes by which HIV-1 enters the mucosal lamina propria [28–33]. Columnar epithelial cells lining the intestinal mucosa can transcytose HIV-1 across the epithelium [28–32]. After entry into the lamina propria, HIV-1 may infect and replicate in local mononuclear target cells or be transported by DCs to draining lymph nodes. Mucosal DCs also take up HIV-1 inoculated onto the apical surface of the intestinal, as well as vaginal, mucosa and transport it through the mucosa for *trans*-infection of local and systemic lymphocytes as we and others have reported [32–34]. Antibodies and innate factors in breast milk may limit vertical HIV-1 transmission by inhibiting one or more of these steps, but to date such inhibitory activity has not been investigated using primary human tissues and mucosal cells.

We characterized the effect of breast milk and its IgG, IgA and non-Ig constituents from HIV-1-infected Ugandan women on the major mucosal transmission events, including the uptake of HIV-1 by primary human intestinal epithelial cells (IECs), DC translocation of virus through intestinal mucosa, and HIV-1 replication in intestinal tissue *ex vivo*. Breast milk IgG, IgA and non-Ig fractions differentially inhibited the entry, transport and infection in explanted human mucosa, offering an explanation, at least in part, for the relatively limited frequency of MTCT of HIV-1 by the breast milk of HIV-1-infected women, despite daily exposure to the virus.

## Materials and Methods

### Breast milk and fractions

Breast milk was collected from HIV-1-infected Ugandan women and HIV-1-seronegative U.S. women at 4–14 weeks postpartum as described [22]. The Ugandan women were enrolled in the Pathobiology of Breast Milk study in Kampala, Uganda [35]. All samples, protocols and consent procedures were approved by the Institutional Review Board (IRB) of the Uganda Virus Research Institute in Uganda, the U.S. Centers for Disease Control and Prevention, the University of Alabama at Birmingham Institutional Review Board, and the Colorado Multiple Institutional Review Board. All participants provided their written informed consent to participate in the study. Breast milk IgG, IgA and non-Ig fractions were separated as described previously [22]. Milk supernatants that showed toxicity to peripheral blood mononuclear cells (PBMCs) by trypan blue exclusion at 2 or 48 hours were excluded.

### HIV-1 strains

Primary Ugandan subtype A HIV-1 isolates 92UG031 and 92UG037 and Ugandan subtype D HIV-1 isolate 92UG005 were obtained from the AIDS Research and Reference Reagent Program, Division of AIDS, NIAID, NIH: HIV-1 from the UNAIDS Network for HIV Isolation and Characterization. These primary virus stocks were expanded in PHA-activated human PBMCs as described [36]. Virus concentration was determined by p24 ELISA (Perkin Elmer, Waltham, MA).

### Isolation of primary human intestinal epithelial cells (IECs)

Human intestinal (jejunal) tissues were obtained from otherwise healthy subjects undergoing gastric bypass for obesity in accordance with IRB approved protocol. IECs were isolated from tissue segments by enzyme digestion, as described previously [28], and purified by depletion of CD3^+^ T cells using anti-CD3 Microbeads (Miltenyi Biotec).

### HIV-1 uptake by IECs

Primary Ugandan subtype A or D HIV-1 viruses (15 ng p24) were pre-incubated with breast milk, IgG, IgA, or the non-Ig fraction for 30 min and applied to suspension of cultured 4 x 10^5^ human primary IECs in duplicate. After 2 hr incubation at 37°C, IECs were washed with complete media three times to remove unbound virus, resuspended in 250 μL of media, frozen and thawed twice. IEC uptake of HIV-1 was then determined by p24 ELISA. HIV-1-specific IgG1 b12 antibody to the CD4 binding site on gp120, polyclonal IgG antibody from pooled normal human serum (Sigma-Aldrich), polyclonal IgA antibody from human colostrum (Sigma-Aldrich), and human cluster II IgA mAb to gp41 membrane proximal external region 2F5 monomeric IgA (mIgA) [32] were used as controls. Breast milk inhibition of HIV-1 uptake by IECs was expressed as % inhibition, with the uptake efficiency of media control defined as 100%. All experiments were performed using tissues from a minimum of 3 different donors for each data point.

### Intestinal mucosal explant and DC uptake and transport of HIV-1 through explanted mucosa

To measure the effect of breast milk and milk fractions on the uptake and transport of HIV-1 by DCs through explanted mucosa, leak-proof explants of intestinal mucosa were constructed as described previously [32, 34, 37]. Primary Ugandan subtype A HIV-1 isolate 92UG031 (45 ng p24) was pre-incubated with breast milk, milk IgG, IgA, or the non-Ig fraction (diluted 1:2) for 30 min, and the mixtures were applied to the apical surface of fresh intestinal mucosal explants. After 2 hr incubation, cells in the lower chamber of the explant system were harvested and (1) analyzed by flow cytometry for DCs that contained HIV-1 using KC57-FITC (an antibody against HIV-1 core antigens) intracellular staining, and CD13-allophycocyanin (APC) and CD11c- phycoerythrin (PE) surface staining, as we previously described [27, 34], or (2) evaluated for HIV-1 by p24 ELISA. Breast milk inhibition of HIV-1 uptake and transport through explanted mucosa was expressed as % inhibition, with the efficiency of media control defined as 100%.

### HIV-1 Infection of intestinal mucosa

After 2 hr incubation of breast milk or milk fractions with HIV-1 on the apical surface of the mucosal explant, the explants were disassembled, and mucosa was washed, treated with trypsin for 10 min at 37°C and washed x3 with PBS to remove virions that remained on the mucosa surface. The tissue was weighed and cultured at 37°C for 3 days. The culture supernatant was harvested to quantify HIV-1 infection of intestinal tissue by p24 ELISA. The HIV-1 reverse transcriptase inhibitor nevirapine was used at a final concentration of 10 μM. Breast milk inhibition of HIV-1 replication was expressed as % inhibition, with the replication level in media control defined as 100%.

### Statistical Analysis

Data are expressed as mean ±SD, and statistical significance between groups was determined using the non-parametric Mann-Whitney test. *p* values ≤ 0.05 were considered significant.

## Results

### Characteristics of breast milk donors

Breast milk was collected from 8 Ugandan women infected with HIV-1 subtype A and 5 HIV-1-seronegative U.S. women between 4 to 10 weeks postpartum. The characteristics of the milk donors are summarized in Table 1. The Ugandan women were a subset of the cohort enrolled in the Pathobiology of Breast Milk study in Kampala, Uganda [35]. Breast milk HIV-1 RNA was undetectable (<50 copies/mL) in 3 mothers and very low with a mean of 77 copies/mL (range 55–714) in the remaining 5. Virus was not cultivable from any of the breast milks. None of the mothers had received antiretroviral therapy other than a perinatal single dose of nevirapine, levels of which were undetectable in breast milk and serum by 4 weeks post-partum [35]. The 5 healthy U.S. mothers had no underlying disease or risk for HIV-1 infection and were not receiving immunosuppressive therapy.

**Table 1:** Characteristics of breast milk donors.

	HIV-1-positive (n = 8)	HIV-1-negative (n = 5)
Average age of mothers (yrs)	25 (range 20–30)	34(range 22–37)
CDC/WHO clinical stage	I	Not applicable
Virus type that infected donors	Subtype A strain	Not applicable
Media plasma HIV-1 RNA	11,336 copies/μL (range <400–290,711)	Not measured
Antiretroviral therapy	Single dose of nevirapine at birth	None
Symptomatic breast illness	No	No
Time of milk collection(Days postpartum)	4–10 weeks	4–10 weeks
Median CD4^+^ T cell count	522 cells/μL (range 332–934)	Not measured
Breast milk IgG (μg/mL)	160.9 ±30.3	39.2 ±7.3
Breast milk IgA (μg/mL)	127.3 ±17.4	206.7 ±30.9

For the assays with skimmed milk (fat layer removed), we used breast milk samples from all 8 HIV-1-infected and the 5 healthy uninfected mothers. For the assays with milk fractions, we purified the IgG, IgA and non-Ig fractions from breast milk of 4 HIV-1-infected women and 3 healthy mothers, due to the limited size of intestinal tissue and the availability of breast milk. Total IgG and IgA in each milk fraction were adjusted to pre-purification levels. The levels of total IgG were significantly higher in the milks from HIV-1-infected women compared with those from HIV-1-negative U.S. women (*p* = 0.02, Table 1), consistent with results in milk from HIV-1-infected and seronegative women in Botswana [38]. In contrast, total IgA was comparable in our two groups (*p* = 0.095, Table 1) but higher in HIV-1-infected women in Botswana. The purified milk IgG, IgA and non-Ig fractions contained undetectable, or barely detectable, Igs of other isotypes (0–0.6%).

### HIV-1-specific IgG and non-Ig components of breast milk inhibit HIV-1 uptake by IECs

**Fig 1:**
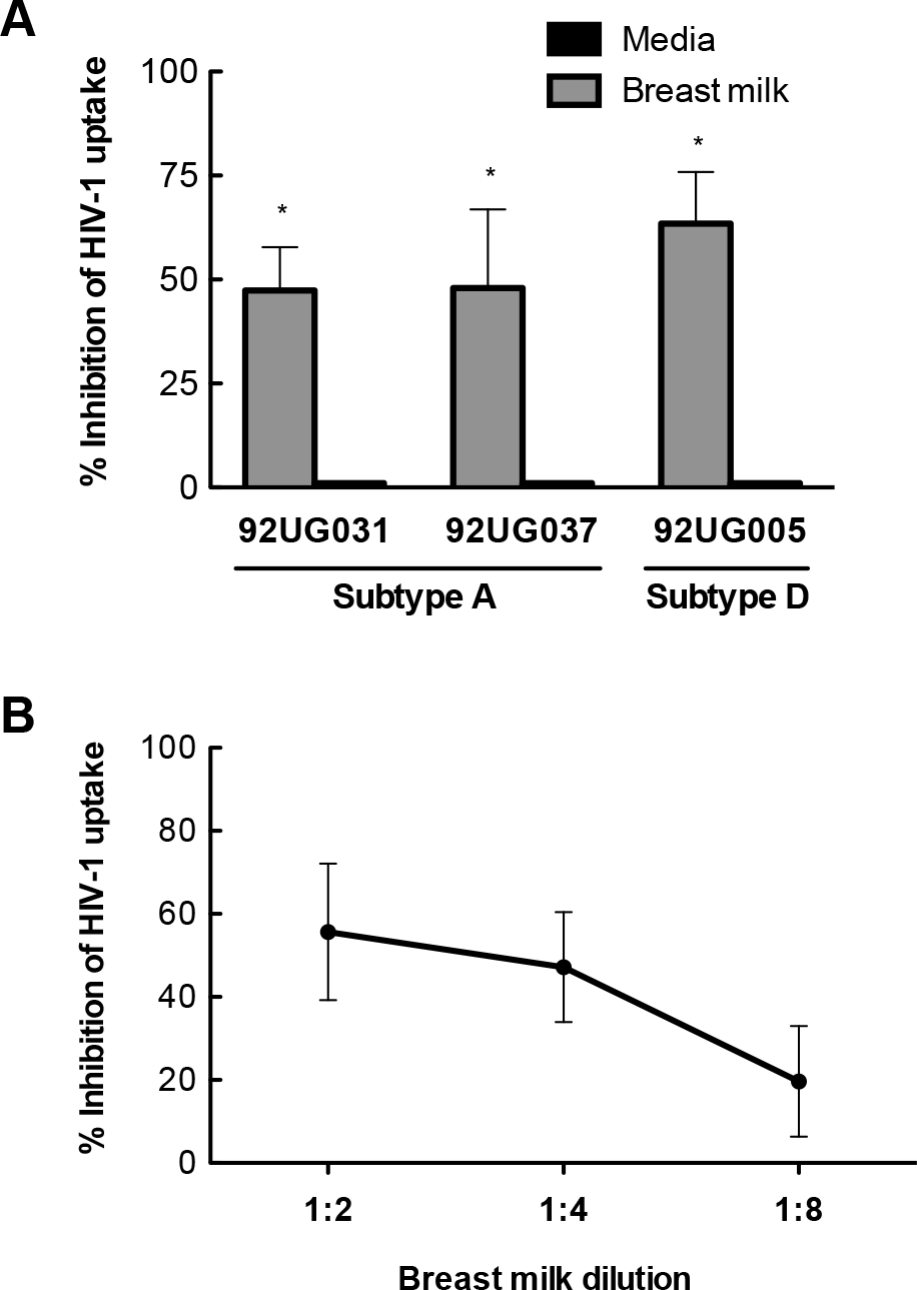
Inhibition of HIV-1 uptake by primary human intestinal epithelial cells (IECs). (**A**) Breast milk inhibition of subtype A and D HIV-1 uptake by IECs. (**B)** Dose-dependent breast milk inhibition of IEC uptake of subtype A HIV-1 by breast milk from an HIV-1-infected Ugandan woman. Ugandan subtype A or D viruses were pre-incubated with breast milk from an HIV-1-infected Ugandan woman for 30 min and then incubated with isolated primary IECs for 2 hr. The uptake of virus by IECs was measured by p24 ELISA with the uptake of virus pre-incubated with media defined as 100%, i.e. no inhibition. The range of p24 in samples treated with breast milk was 622–1300 pg/mL. Results are the mean values ±SD using IECs isolated from 4 separate tissue donors. Differences in IEC uptake of virus pre-incubated with breast milk and virus pre-incubated with media was determined by non-parametric Mann-Whitney test with significance indicated by * (*p* < 0.05).

The upper gastrointestinal tract is the portal through which HIV-1 enters the host in the majority of MTCTs [1, 2, 6]. After ingestion and passage into the small intestine, HIV-1 in breast milk initially encounters IECs and, possibly, DCs. Therefore, we first determined the ability of breast milk and its components to block HIV-1 binding to and uptake by IECs, the first steps in the transcytosis process. A representative breast milk (BM5) from an HIV-1-infected Ugandan woman markedly inhibited IEC uptake of subtype A isolates 92UG031 and 92UG037, as well as subtype D isolate 92UG005, the subtypes that represent the majority of strains in Uganda (Fig 1A). The subtype A isolate 92UG031 was used in the subsequent experiments. Next, we showed that BM5 inhibited the uptake of subtype A virus 92UG031 by IECs in a dose-dependent manner (Fig 1B). Based on the dose-curve study, the mid-point dilution (1:4) was selected for the following IEC uptake assays in order to limit any toxicity and to help standardize pH, osmolarity and nutrients between samples.

**Fig 2:**
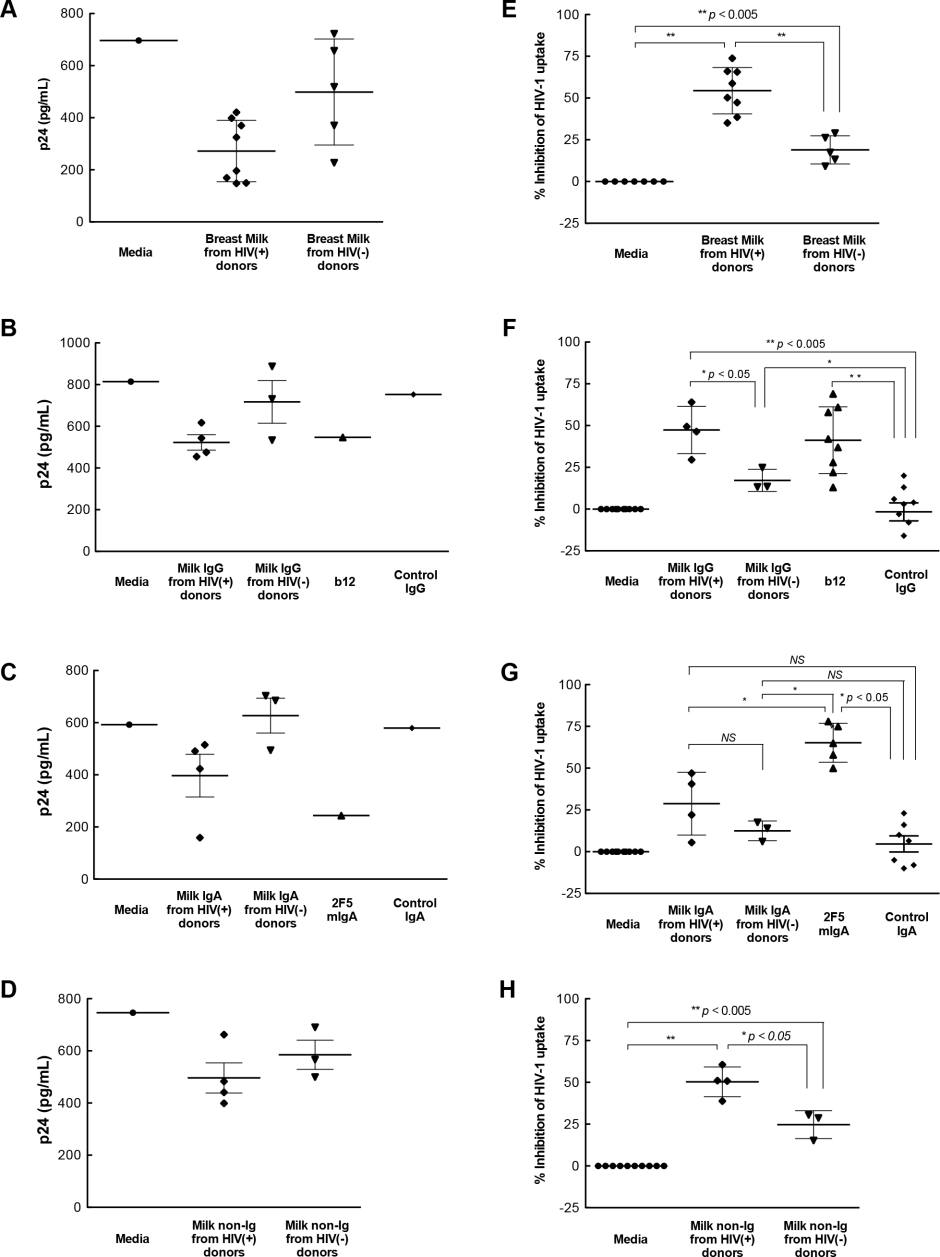
Inhibition of IEC uptake of HIV-1. Ugandan subtype A HIV-1 was pre-incubated with **(A, E)** breast milk or the corresponding **(B, F)** IgG, **(C, G)** IgA or **(D, H)** non-Ig components (diluted 1:4) and then incubated with isolated primary IECs for 2 hr. IEC uptake of virus was measured by p24 ELISA in homogenized IEC. Results in panels **A-D** are p24 values from a representative tissue donor. Values in panels **E-H** are the mean percent inhibition using IECs isolated from 3–5 tissue donors for each breast milk. The uptake of virus pre-incubated with media was defined as 100%, i.e. no inhibition. For control antibodies, each dot point represents a mean value from a separate tissue donor. Control IgG: human serum polyclonal IgG. b12: human IgG1 antibody against the CD4 binding site on gp120. Control IgA: human colostrum polyclonal IgA. 2F5 mIgA: Human cluster II monomeric IgA mAb to gp41 membrane proximal external region. Differences in the uptake for virus pre-incubated with breast milk, media or milk component was determined by the non-parametric Mann-Whitney test with significance indicated by *NS* (not significant), * (*p* < 0.05), or ** (*p* < 0.005).

Breast milk from both HIV-1-infected Ugandan women and HIV-1-negative women (diluted 1:4) significantly inhibited IEC uptake of subtype A isolate 92UG031 compared with virus pre-incubated with media (Fig 2A and 2E). Breast milk from HIV-1-infected Ugandan women inhibited IEC uptake of HIV-1 by a mean of 54% (*p*<0.005), whereas breast milk from HIV-1-negative women inhibited uptake by 19% (*p*<0.005). Thus, skimmed breast milk for both HIV-1-infected and uninfected women significantly inhibited virus uptake by IECs, but milk from infected women was significantly more effective in limiting uptake.

Both antibodies and innate factors in breast milk may contribute to neutralization and protection against the postnatal transmission of HIV-1 from mother to child [13–15, 19, 21, 39, 40]. Therefore, we examined which of these components contributed to whole breast milk inhibition of HIV-1 uptake by IECs. The IgG fractions derived from breast milk of both HIV-1-infected and uninfected women significantly inhibited IEC uptake of subtype A virus 92UG031 by a mean of 47% (*p*<0.005) and 17% (*p*<0.005), respectively, as did IgG1 b12 antibody (37%; p<0.0005) (Fig 2B and 2F) compared with media or IgG isotype control. Inhibition of virus uptake by IECs was significantly higher with milk from infected than control women (p<0.05). In contrast, breast milk IgA fractions from HIV-1-infected and uninfected women inhibited IEC uptake of HIV-1 at similar levels to that of isotype control IgA (Fig 2C and 2G). Thus, although IgG in breast milk from both groups of women showed some activity, inhibition was greater with IgG isolated from HIV-1-infected women. In contrast, milk IgA had no such effect.

In addition to the effects of breast milk IgG, milk non-Ig fractions from both HIV-1-infected and uninfected women inhibited IEC uptake of subtype A virus 92UG031 by a mean of 50% and 25%, respectively, compared with media control (*p*<0.005). Notably, the non-Ig fractions from infected women more potently inhibited IEC uptake than that from uninfected women (*p*<0.05) (Fig 2D and 2H). Thus, the ability of breast milk to inhibit the uptake of HIV-1 by primary IECs was associated with the presence of HIV-1 infection in the donor and was mediated by both milk IgG and non-Ig components.

### Blockage of the uptake and transport of HIV-1 through intestinal mucosa

**Fig 3:**
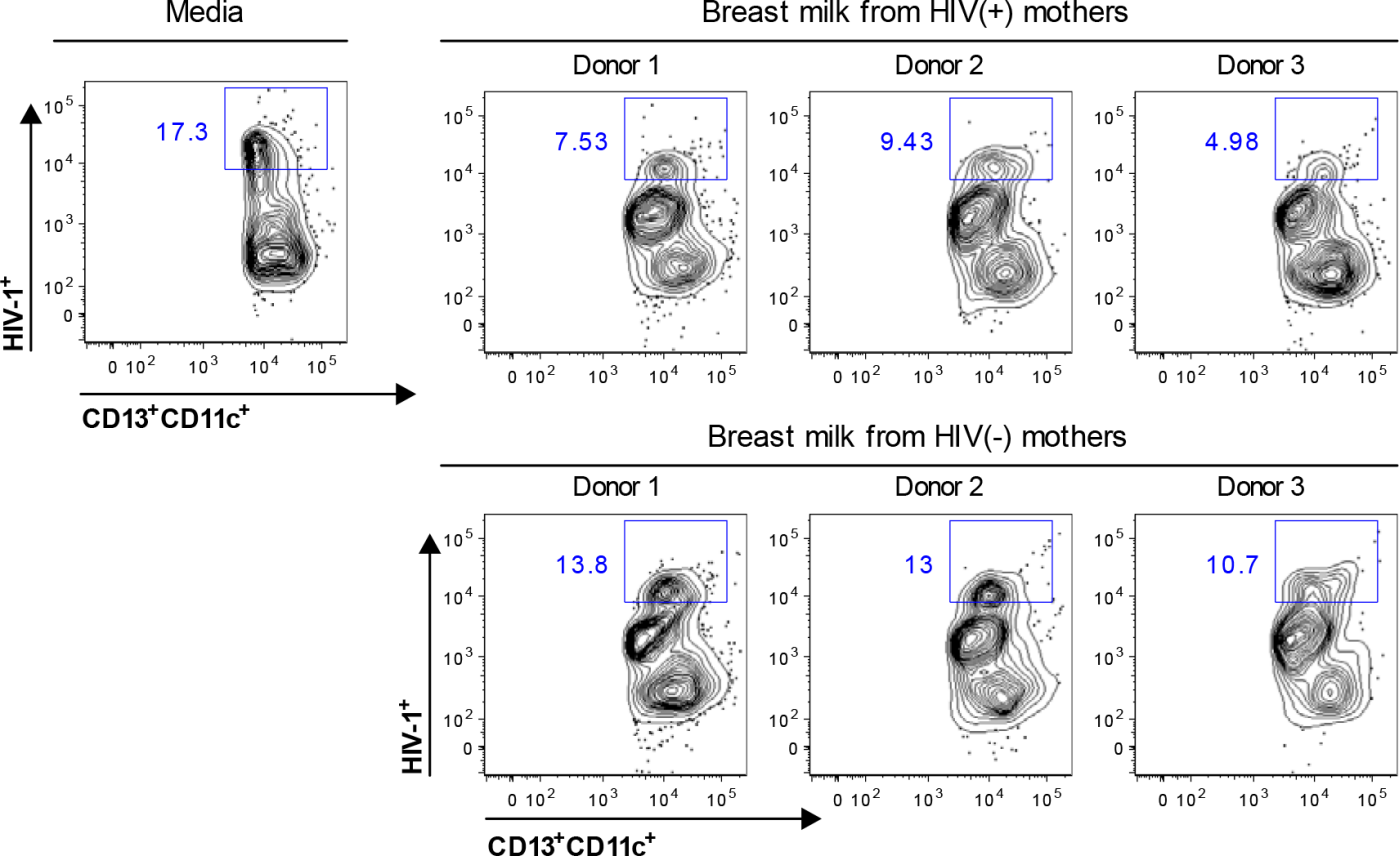
Breast milk impacts myeloid DC uptake and transport of HIV-1 through intestinal mucosa. Ugandan subtype A HIV-1 was pre-incubated with skimmed breast milk (diluted 1:2) for 30 min, applied to the apical surface of explanted intestinal mucosa. After 2 hr, cells in the lower chamber of the explant system were harvested and analyzed by flow cytometry by gating on myeloid DCs (CD13^+^CD11c^+^) that contained HIV-1 using KC57-FITC. Results are representative of experiments with two tissue donors.

**Fig 4:**
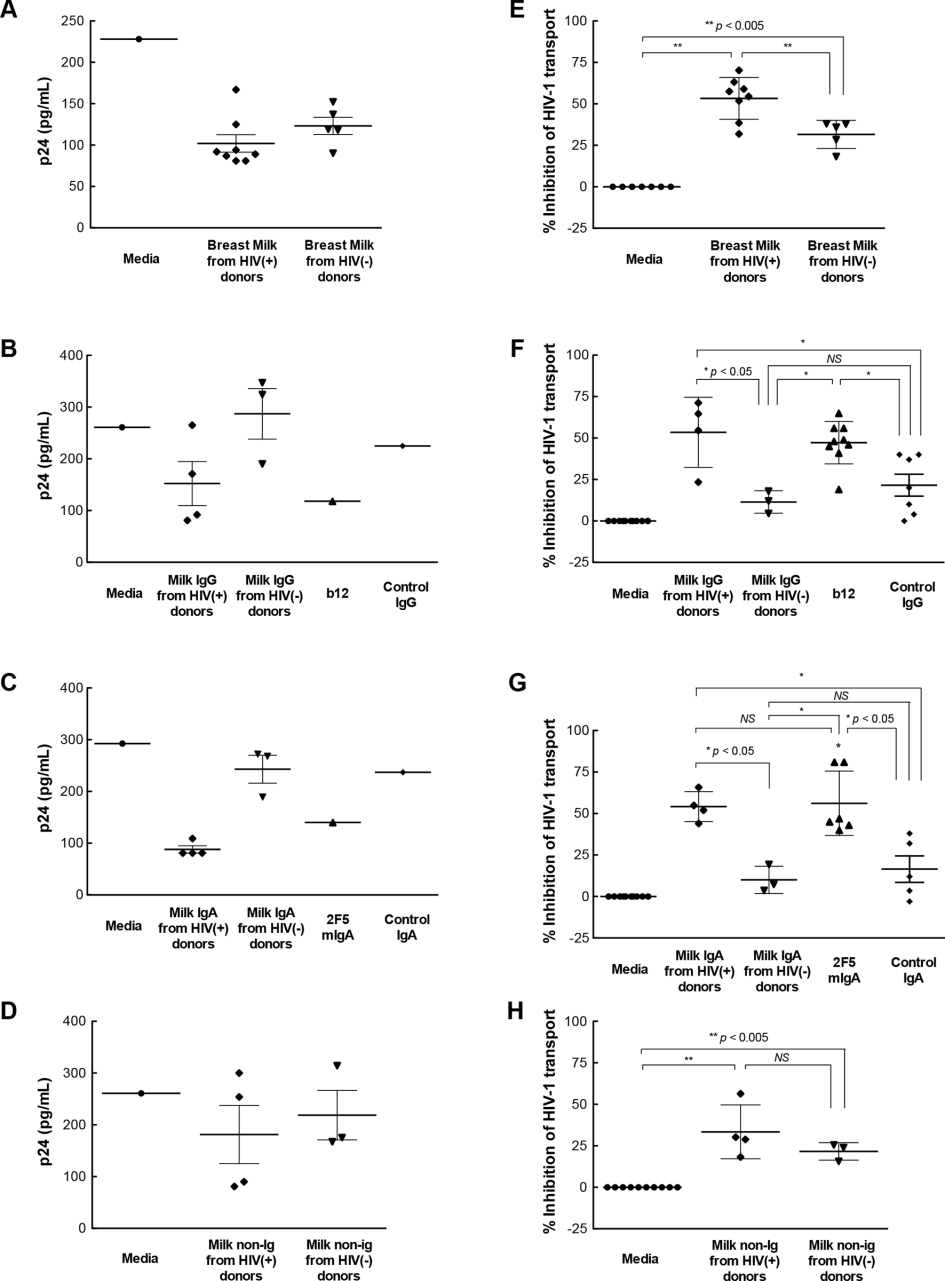
Impact on myeloid DC uptake and transport of HIV-1 through intestinal mucosa. Ugandan subtype A HIV-1 was pre-incubated with **(A, E)** skimmed breast milk or the corresponding **(B, F)** IgG, **(C, G)** IgA or **(D, H)** non-Ig component (diluted 1:2) for 30 min, applied to the apical surface of explanted intestinal mucosa, and 2 hr later cells in the lower chamber of the explant system were harvested, lysized and measured for HIV-1 by p24 ELISA. Results in panels **A-D** are p24 values from a representative tissue donor. Values in panels **E-H** represent the mean percent inhibition of HIV-1 transport by one breast milk through tissue from 3–5 separate tissue donors for unfractionated milk (**E**), purified milk IgG (**F**), purified milk IgA (**G**), and Non-Ig fractions (**H**). DC uptake and transport of virus pre-incubated with media was defined as 100%, i.e. no inhibition. For control antibodies, each dot point represents a mean value from a separate tissue donor. Differences in HIV-1 transport through the mucosa are noted by *NS* (not significant), * (*p* < 0.05), or ** (*p* < 0.005).

We have shown that within two hours of inoculation onto the apical surface of the mucosa, only myeloid DCs take up HIV-1, transport captured virus through the mucosa, and then transmit the virus in trans to peripheral blood and mucosal lymphocytes [32, 34]. Therefore, we investigated whether breast milk blocks myeloid DC capture and mucosal translocation of HIV-1 using our leak-proof intestinal explant system, in which the integrity of epithelial tight junctions was maintained for at least 2 hr incubation [32, 41]. Primary Ugandan subtype A HIV-1 isolate 92UG031 was pre-incubated with breast milk (diluted 1:2) for 30 min, and the mixtures were applied to the apical surface of fresh intestinal mucosal explants. After 2 hr incubation, cells in the lower chamber of the explant system were harvested and analyzed by flow cytometry for myeloid DCs that contained HIV-1 using KC57-FITC, CD13-APC and CD11c-PE antibodies. Breast milk from HIV-1-infected Ugandan women and, to a lesser extent, HIV-1-seronegative women decreased the percentage of myeloid DCs (CD13^+^CD11c^+^) that contained HIV-1 and migrated into the lower chamber when compared with that of media control (Fig 3). The inhibition of percentage of myeloid DCs (CD13^+^CD11c^+^) that captured HIV-1 and migrated into the lower chamber was similar for two separate tissue donors (data not shown). These data suggest that breast milk inhibits myeloid DC capture and transport of subtype A virus through human small intestinal mucosa. To further quantitatively examine whether breast milk inhibits the total HIV-1 captured and transported by myeloid DCs through intestinal mucosa, the cells in the lower chamber of the explant system were harvested and the amount of virus transported by the cells was quantified by p24 ELISA (Fig 4A). Breast milk from HIV-1-infected Ugandan women inhibited the capture and transport of subtype A virus through intestinal mucosa by 53% (*p*<0.005) (Fig 4A and 4E). Breast milk from healthy HIV-1-seronegative women also inhibited the uptake and translocation of HIV-1 by a mean of 32% compared with that of media alone (*p*<0.005), albeit less effectively than milk from HIV-1-infected women (*p*<0.05). Together, these findings suggest that HIV-1-specific antibodies and innate factors inhibit myeloid DC uptake and transport of virus through intestinal mucosa.

We next examined the ability of breast milk IgG, IgA and non-Ig components to individually inhibit the uptake and transport of HIV-1 through intestinal mucosa. The IgG fraction of breast milk from HIV-1-infected Ugandan women inhibited the uptake and transport of virus through intestinal mucosa by 53%, well above that of IgG from uninfected women (11%, p<0.05) and control IgG (22%, *p*<0.05) (Fig 4B and 4F). The IgG and IgA fractions of breast milk from HIV-1-infected women displayed levels of inhibition equivalent to those of unfractionated breast milk, control IgG1 b12 and 2F5 mIgA antibodies (Fig 4B and 4C and 4F and 4G). Similar to the inhibitory activity of IgG, the IgA fraction also inhibited uptake and transport of virus through the intestinal mucosa compared with media alone (54%, *p*<0.05), isotype IgA control (22%) and IgA from healthy mothers (10%) (Fig 4C and 4G). The breast milk IgG and IgA fractions from uninfected women displayed levels of inhibition similar to those of the corresponding isotype control antibodies (Fig 4F and 4G). The level of inhibition by the non-Ig fraction of milk from HIV-1-infected and healthy women was similar, with a mean inhibition of 33% and 24%, respectively (Fig 4D and 4H). Thus, HIV-1-specific IgG and IgA antibodies in breast milk suppressed the uptake and transport of HIV-1 through intestinal mucosa.

### Inhibition of HIV-1 infection in human intestinal mucosal tissue

**Fig 5:**
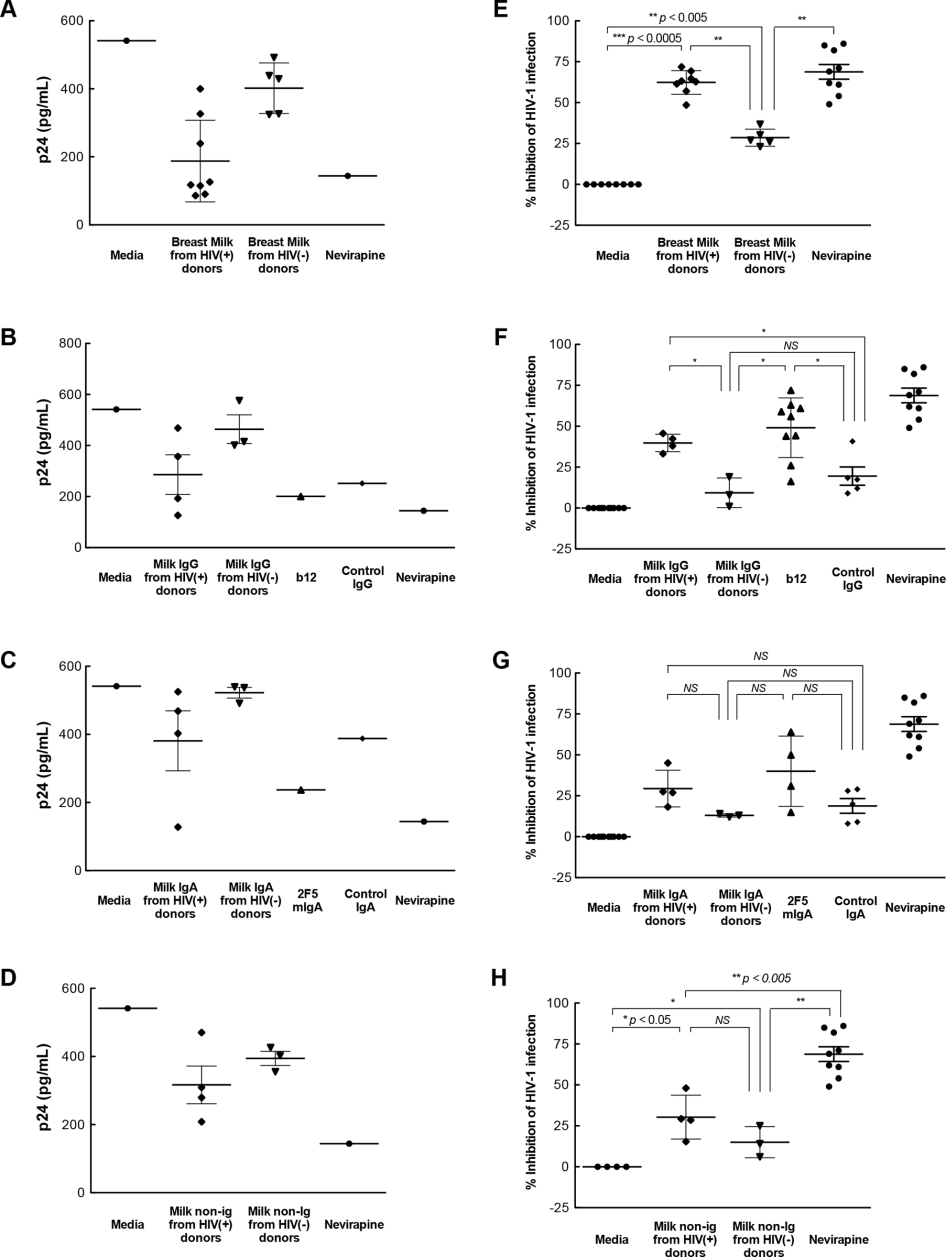
HIV-1 replication in intestinal mucosa. Ugandan subtype A HIV-1 was pre-incubated with **(A, E)** breast milk or the corresponding **(B, F)** IgG, **(C, G)** IgA or **(D, H)** non-Ig components (diluted 1:2) for 30 min and inoculated onto the apical surface of explanted intestinal mucosa. After 2 hr incubation, the mucosa was trypsinized, incubated for 3 days and HIV-1 replication was measured by p24 released into the media normalized to tissue weight. Results in panels **A-D** are p24 values from a representative tissue donor. Values in panels **E-H** represent the mean percent inhibition of HIV-1 infection in intestinal tissue from 3–5 different donors by each breast milk. For control antibodies and Nevirapine control, each dot point represents a mean value from a separate tissue donor. Infection with virus pre-incubated with media was defined as 100%, i.e. no inhibition. Differences in viral replication in the tissue when the virus was pre-incubated with breast milk, media or milk components was determined using the non-parametric Mann-Whitney test, and significance is indicated by *NS* (not significant), * (*p* < 0.05), ** (*p* < 0.005), or *** (*p* < 0.0005).

We next characterized the effect of breast milk and milk fractions on HIV-1 replication in human intestinal mucosa. Incubation of virus with breast milk from HIV-1-infected Ugandan women inhibited viral replication in the mucosa (62%), an effect comparable to inhibition by the reverse transcriptase inhibitor nevirapine (69%) (Fig 5A and 5E). Milk from uninfected women supported a more modest level of inhibition of replication in mucosal tissue (29%), lower than that with milk from HIV-1-infected women (*p*<0.005).

To identify the factors in breast milk capable of inhibiting mucosal HIV-1 replication, we first tested the IgG fraction for inhibitory activity. The IgG fraction from breast milk of HIV-1-infected Ugandan women significantly inhibited HIV-1 mucosal infection by a mean of 40%, similar to that of IgG1 b12 and nevirapine, This activity exceeded that of IgG from healthy women (9%, *p*<0.05) and control IgG (20%, *p*<0.05) (Fig 5B and 5F). Replication of virus in the intestinal mucosa was lower with IgA from HIV-1-infected women (29%), although inhibition was not significantly different from that of IgA from healthy seronegative mothers (13%) or control isotype IgA (19%) (Fig 5C and 5G). The IgG and IgA fractions from healthy women displayed levels of inhibition similar to that of the corresponding isotype control antibodies (Fig 5B and 5C and 5F and 5G). The inhibition by innate factors in the non-Ig fractions of milk from HIV-1-infected and uninfected women was modest (mean 30% and 15%, respectively) (Fig 5D and 5H). In summary, these data suggest that HIV-1-specific IgG in breast milk significantly suppressed HIV-1 infection in primary intestinal mucosal cells and tissue. Breast milk IgA and non-Ig fractions also suppress infection but less effectively, suggesting a complementary role in milk inhibition of vertical transmission. Taken together, our results show that breast milk inhibits several sequential steps, including virus uptake, transport through and infection of intestinal mucosa, in HIV-1 mucosal transmission process.

## Discussion

Among nursing infants exposed daily to HIV-1 in infected breast milk, only a minority (8–15%) of such infants become infected postnatally [2–6], indicating that breast milk acts as a vehicle of protection, as well as a source of transmission. Here, we used human primary intestinal epithelial cells (IECs) and a human intestinal explant system that recapitulates the mucosa *in situ* [32, 37, 41] to demonstrate that breast milk and its components from HIV-1 infected Ugandan women inhibit mucosal uptake, transport and replication of primary subtype A virus.

HIV-1 translocation across intestinal epithelium by epithelial cell uptake followed by transcytosis and/or DC transport is critical for initiating MTCT. We investigated the effect of breast milk on IEC uptake of HIV-1, the first step in the IEC transcytotic process. Using human primary IECs, we show that breast milk from a subtype A HIV-1-infected Ugandan woman inhibited IEC uptake of an Ugandan subtype A isolate, as well as a heterologous subtype A and a subtype D isolate, suggesting that breast milk inhibition of HIV-1 transmission is not restricted to the virus of the infected mother. Furthermore, our results indicate that both IgG, likely the HIV-1-specific IgG antibodies, and non-Ig components, in breast milk contribute to the inhibition of HIV-1 uptake by IECs. In this regard, we previously reported that HIV-1-specific IgG and IgA antibodies inhibit cell-free HIV-1 transcytosis across model epithelium and rectal mucosa *ex vivo* [36, 41]. Transcytosis of cell-associated HIV-1 can be inhibited *in vitro* by dimeric IgA and pentameric IgM isolated from HIV-1-infected subjects [31, 42], secretory IgA against gp41 [43, 44], mucosal and serum IgA from HIV-1-exposed seronegative persons [45, 46], anti-gp160 IgG and secretory IgA [47, 48], and 2G12 [42] in immortalized cell lines, results extended herein with primary human mucosal cells and tissue. Furthermore, antibodies to host cell epitopes such as CCR5 and GalCer can block cell-free HIV-1 transcytosis across model epithelium and primary epithelial cells [28, 48, 49], which may explain, in part, our finding that breast milk and the IgG fraction from uninfected donors partially inhibited HIV-1 uptake by IECs. These results indicate that innate factors in the non-Ig fraction of milk could inhibit HIV-1 uptake by IECs. Thus, breast milk antibodies and innate factors, alone or in combination, may disrupt IEC uptake *in vivo*, limiting mucosal transmission of the virus.

Lamina propria DCs also may capture HIV-1 inoculated onto the apical mucosal surface via dendrites that extend across the epithelium [33, 34, 50] or capture virus transcytosed by epithelial cells into the lamina propria [34, 50]. The DCs then could *trans*-infect local lamina propria CD4^+^ T cells or transport the captured virus through the mucosa to initiate systemic infection [32, 34]. Indeed, we have shown that myeloid DCs are the only cells that mediate uptake and transfer of HIV-1 across mucosal tissue within 2 hr [32, 34]. Build on those results, we show that breast milk from HIV-1-infected women significantly inhibited the uptake and transport of HIV-1 by DCs through intestinal mucosa. This inhibitory activity of breast milk is likely due to a combination of HIV-1-specific IgG, HIV-1-specific IgA, and non-Ig innate factors. Breast milk inhibition of the uptake and transport of HIV-1 through the mucosa could be due to (1) diminished capture of HIV-1 by DC dendrites that extend across the epithelium, (2) reduced DC transport of virus through the tissue, and/or (3) reduced epithelial cell transcytosis of HIV-1, resulting in less virus entering the lamina propria for uptake and transport by DCs.

Breast milk from HIV-1-infected and -uninfected women has been shown to inhibit HIV-1 replication in TZM-bl cells [21, 23, 51–53]. In our report, we show that breast milk from HIV-1-infected women significantly inhibited infection of primary Ugandan subtype A virus in human intestinal mucosa, presumably in susceptible CD4^+^ T cells since intestinal macrophages do not support HIV-1 replication, as we have reported [37, 54]. Up to 85% inhibition of HIV-1 replication in TZM-bl cells has been reported for milk from HIV-1-infected women [53], as well as 65–100% inhibition for milk from HIV-1-seronegative donors [23, 53]. In addition, human breast milk from uninfected women has been reported to markedly reduced oral transmission of HIV-1 in BLT humanized mice [23]. In contrast, we used fresh human intestinal tissue to show that milk from healthy uninfected women inhibited HIV-1 replication in the tissue only modestly (29%), whereas milk from infected women more effectively inhibited replication (62%) in intestinal mucosa. HIV-1-specific IgG is likely the major contributor to the inhibition. The apparent difference may be explained by the model systems (*ex vivo* mucosal tissue versus *in vitro* cell line cells), the virus isolates, and whole versus skim breast milk. Furthermore, our preliminary studies show that this inhibition is, at least partially, due to neutralizing antibodies, because breast milk from HIV-1-infected women with neutralizing activity supported significantly higher inhibitory activity in the intestinal mucosa model compared with non-neutralizing breast milk (data not shown).

In summary, we report that antibodies and innate factors in breast milk inhibit the uptake, transport, and replication of HIV-1 in human intestinal mucosa, key steps in the pathogenesis of primary HIV-1 infection of infants by breastfeeding. These factors likely serve complementary functions *in vivo*, acting in concert to limit vertical HIV-1 transmission. Further studies categorizing breast milk from HIV-1-infected mothers into those that do and do not neutralize the virus, and those from transmitting and non-transmitting mothers are warranted. The model systems described herein provide relevant mechanisms to identify protective components in breast milk that can be utilized individually or in combination to prevent post-partum transmission of HIV-1 in the small intestine, and, potentially, other mucosal sites. Our data also suggests that a successful preventive or therapeutic approach would require multiple immune factors functioning at multiple steps of HIV-1 mucosal transmission.
